# Structural equation modeling for hypertension and type 2 diabetes based on multiple SNPs and multiple phenotypes

**DOI:** 10.1371/journal.pone.0217189

**Published:** 2019-09-12

**Authors:** Saebom Jeon, Ji-yeon Shin, Jaeyong Yee, Taesung Park, Mira Park

**Affiliations:** 1 Department of Marketing Information Consulting, Mokwon University, Daejeon, KOREA; 2 Department of Preventive Medicine, School of Medicine, Kyungpook National University, Daegu, KOREA; 3 Department of Physiology and Biophysics, Eulji University, Daejeon, KOREA; 4 Department of Statistics, Seoul National University, Seoul, KOREA; 5 Department of Preventive Medicine, Eulji University, Daejeon, KOREA; GeneDx, UNITED STATES

## Abstract

Genome-wide association studies (GWAS) have been successful in identifying genetic variants associated with complex diseases. However, association analyses between genotypes and phenotypes are not straightforward due to the complex relationships between genetic and environmental factors. Moreover, multiple correlated phenotypes further complicate such analyses. To resolve this complexity, we present an analysis using structural equation modeling (SEM). Unlike current methods that focus only on identifying direct associations between diseases and genetic variants such as single-nucleotide polymorphisms (SNPs), our method introduces the effects of intermediate phenotypes, which are related phenotypes distinct from the target, into the systematic genetic study of diseases. Moreover, we consider multiple diseases simultaneously in a single model. The procedure can be summarized in four steps: 1) selection of informative SNPs, 2) extraction of latent variables from the selected SNPs, 3) investigation of the relationships among intermediate phenotypes and diseases, and 4) construction of an SEM. As a result, a quantitative map can be drawn that simultaneously shows the relationship among multiple SNPs, phenotypes, and diseases. In this study, we considered two correlated diseases, hypertension and type 2 diabetes (T2D), which are known to have a substantial overlap in their disease mechanism and have significant public health implications. As intermediate phenotypes for these diseases, we considered three obesity-related phenotypes—subscapular skin fold thickness, body mass index, and waist circumference—as traits representing subcutaneous adiposity, overall adiposity, and abdominal adiposity, respectively. Using GWAS data collected from the Korea Association Resource (KARE) project, we applied the proposed SEM process. Among 327,872 SNPs, 24 informative SNPs were selected in the first step (p<1.0E-05). Ten latent variables were generated in step 2. After an exploratory analysis, we established a path diagram among phenotypes and diseases in step 3. Finally, in step 4, we produced a quantitative map with paths moving from specific SNPs to hypertension through intermediate phenotypes and T2D. The resulting model had high goodness-of-fit measures (χ^2^ = 536.52, NFI = 0.997, CFI = 0.998, GFI = 0.995, AGFI = 0.993, RMSEA = 0.012).

## Introduction

Hypertension and type 2 diabetes (T2D) are two of the leading risk factors for atherosclerotic cardiovascular disease, which is a major component of the global burden of disease [1–4]. These conditions often occur together, and recent studies showed that the presence of T2D increased the risk of hypertension [5, 6]. Hypertension and T2D are thought to share common pathways such as obesity, insulin resistance, inflammation, oxidative stress, and mental stress [7]. In addition to lifestyle and environmental factors, genetic factors have also been explored to understand the mechanisms of T2D and hypertension [7, 8]. Because obesity-related phenotypes are thought to be a common pathophysiological element underlying T2D and hypertension, understanding the connections among these diseases and factors related to obesity is an important aspect of the search for proper treatments of these diseases.

Genome-wide association studies (GWAS) have been successful in identifying genetic variants associated with complex diseases such as asthma, autism disorder, T2D, and hypertension [9]. A typical approach of GWAS is to report significant single-nucleotide polymorphisms (SNPs) affecting disease; in other words, such studies generally present single-SNP analyses. However, association analyses between genotypes and traits are not straightforward due to the complex relationships between genetic and environmental factors. Moreover, in the presence of multiple correlated phenotypes and/or diseases, such analyses become more complicated. Since a separate analysis of each phenotype or disease ignores dependencies among phenotypes, a multivariate approach considering a joint analysis should be considered. Various genomic studies have been conducted to understand hypertension and T2D individually [10, 11]. However, few studies have attempted to model the pathways underlying hypertension through obesity-related traits and T2D [12].

Methodology for mediation to assess the importance of various paths and mechanisms has expanded rapidly in the last few decades [13]. Mediation analysis has traditionally been dealt with in the fields of social sciences and psychology, but more recently it has also been addressed in the fields of epidemiology and health. Mediation processes are framed in terms of intermediate variables, the mediator, that helps explain how or why an independent variable influences an outcome. The mediator significantly accounts for variation in an outcome variable [14]. In the context of mediation analysis, there are many advantages in using the structural equation model (SEM) framework [15]. SEM is a multivariate statistical method that involves the estimation of parameters for a system of simultaneous equations [16]. It can be used when extending a mediation process to multiple independent variables, mediators, or outcomes.

In this study, we present SEM-based approach to studying the associations between genetic variants and phenotypes. In general, SEM is a confirmatory approach rather than an exploratory approach, in that the main focus of SEM is to verify that a model established by a researcher beforehand is supported by the data [17], although it may sometimes seem exploratory since the model can be modified to improve its goodness-of-fit. SEM implies a functional relationship expressed through a conceptual model, path diagram, and mathematical equations. Therefore, it is possible to express, through SEM, the causal relationships in a hypothesized mediation process, the simultaneous nature of the indirect and direct effects, the dual role the mediator plays as both a cause and an effect [15, 18]. Various types of models can be used in SEM, including regression, path, confirmatory factor, and growth curve models [19].

SEM has been used in various fields, including genetic analysis [20, 21]. Procedures for applying SEM after gene- and pathway-based analysis have been proposed [22]. Also, a method for merging GWAS and gene regulatory networks (GRNs) using the SEM framework has been attempted [23]. More recently, the GW-SEM method, which relies on a diagonally weighted least squares (DWLS) estimator, has been proposed to construct SEM on a genome-level [24]. The SEM-based genome scan approach for metabolic syndrome has been carried out [25]. Additionally, cross-sectional data analysis to provide a path diagram from genetic variants to metabolic syndrome and disease has been studied. SNPs with significant associations with phenotypic traits were as exogenous predictors [8]. Meanwhile, the approaches to analyzing longitudinal data with pedigrees have also been developed [26, 27]. However, there are few studies that have applied SEM to analyze multiple SNPs, intermediate phenotypes, diseases simultaneously.

In this respect, we suggest a procedure for constructing an SEM to investigate the relationships of multiple SNPs and multiple intermediate phenotypes with respect to multiple diseases. Unlike current methods that focus only on identifying direct associations between diseases and SNPs, our method introduces the effects of intermediate phenotypes. We define intermediate phenotypes as disease-related phenotypes distinct from the target itself. Moreover, we simultaneously consider multiple diseases in the model. As a result, we generated a quantitative map simultaneously showing the relationships among multiple SNPs, phenotypes, and diseases.

Our detailed goals were to answer the following research questions. First, do genetic characteristics such as SNPs affect intermediate phenotypes and/or diseases? Second, is there any commonality or similarity between the SNPs? Furthermore, if there is a genetic commonality, can we consider their underlying component, by taking that commonality into account? How do such components affect intermediate phenotypes and disease? Third, are there any associations or causal relationships between the intermediate phenotypes and diseases under consideration? Fourth, is it possible to simultaneously consider the relationships of multiple SNPs, intermediate phenotypes, and diseases? To answer these questions, we developed an SEM-based procedure that introduced intermediate phenotypes to examine both direct and indirect relationships. We applied this approach to Korean GWAS data. We considered two diseases (hypertension and T2D) and three intermediate variables related to obesity (subscapular skin fold thickness [SUB], body mass index [BMI], and waist circumference [WC]). Using Korean GWAS data, we constructed a model with paths extending from genetic variants to hypertension through obesity-related intermediate phenotypes and T2D.

## Materials and methods

### SEM-based modeling procedure of multiple SNPs and multiple phenotypes

**Fig 1:**
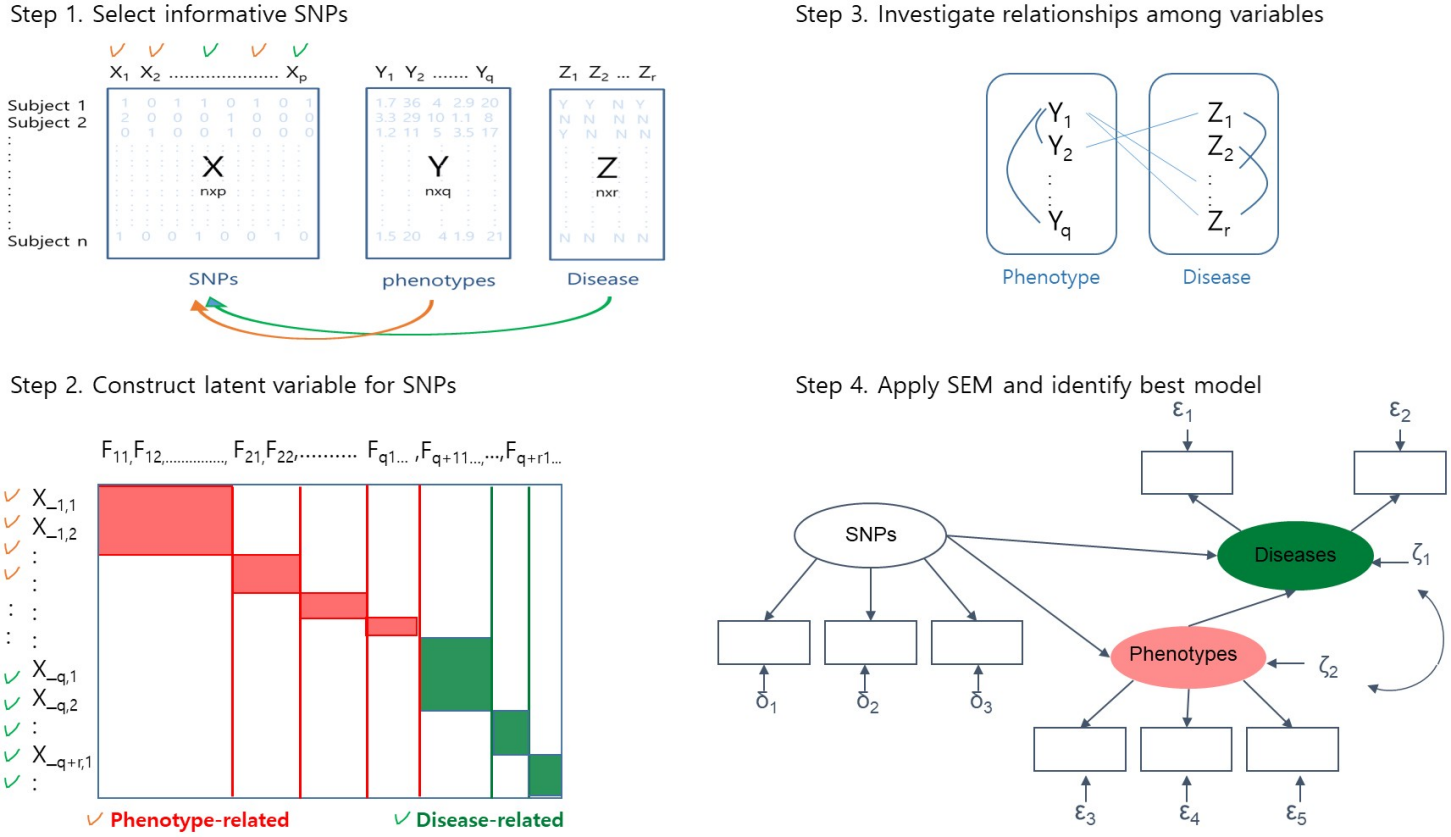
Summary of SEM-based modeling procedures for genomic data. *F*_*ij*_ represents the j^th^ factor loading for the i^th^ SNP block. X_i,k represents the k^th^ SNP in the i^th^ SNP block.

Consider an n×c data matrix A with three blocks for n samples: A = [X|Y|Z]. The first block X contains data from n×p SNPs, Y is a block corresponding to n×q intermediate phenotypes, and Z is a block corresponding to n×r diseases, where c = p+q+r. In order to investigate the multiple phenotypes that reflect joint action of multiple SNPs, we developed an SEM-based modeling procedure summarized in the following four steps. Fig 1 shows a summary of the proposed procedure.

The first step was to select the preliminary informative SNPs. To avoid computational complexity and multicollinearity due to the enormous scale of the SNPs in GWAS, non-contributing SNPs to each phenotype were excluded through a single-SNP analysis using regression or logistic regression models. Since intermediate phenotypes and diseases are likely to be heterogeneous according to demographic factors such age and sex, analyses were conducted using demographic factors as covariates. Instead, it is possible to choose the most significant SNP in each linkage disequilibrium (LD) block. In this paper, a set of significant SNPs for each phenotype or disease will be called SNP block. Thus, q+r SNP blocks consisting of informative SNPs are produced in this step.

**Fig 2:**
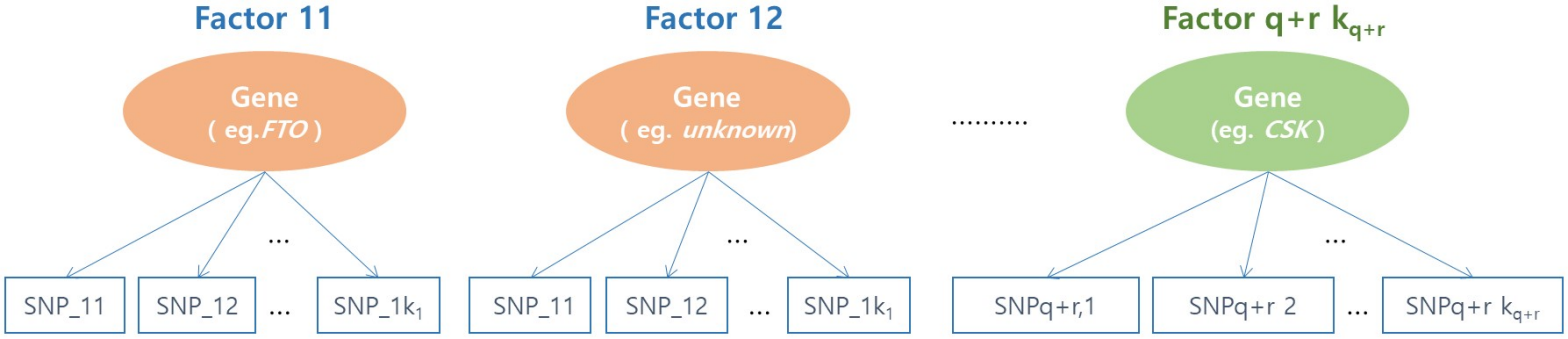
Conceptual framework for exploratory factor analysis. Factor ij represents the j^th^ latent variable for i^th^ SNP block. SNP_ik represents the k^th^ SNP in i^th^ SNP block.

The second step was to explore the underlying nature of the factors, or latent variables of the selected SNPs for each SNP block. We begin with theoretical assumption that there exists a common latent construct among the significant SNPs. Fig 2 shows a conceptual framework for the factor analysis. We assumed that SNPs having similar genetic functions or located near the same gene are manifested by the underlying latent factors. In the exploratory factor analysis steps, we performed a procedure of varimax rotation, which is one of the most common method of orthogonal rotation, producing uncorrelated factors and simplified interpretation [28]. In order to construct latent variables efficiently, SNPs with a very low communality were excluded. Here, communality was considered to represent the variance of any SNP that was shared with other SNPs via common factors.

The third step was to investigate the effect of intermediate phenotypes on diseases, or the associations among all these phenotypic variables. This association analysis of multiple phenotypes provided a conceptual framework for constructing an SEM structure in the following step.

In the final step, we applied SEM based on the previously constructed latent variables, or joint SNPs, obtained from step 2. The SEM reflected the relationships between all SNPs, their joint action or latent variables, intermediate phenotypes, disease, and morbidity. A typical process for SEM was performed [13]. That is, after identification of the model, we estimated and tested the model.

Because of the distribution issue of the indirect effect, several approaches have been suggested to test the unstandardized indirect effect [29]. Bootstrapping approach uses the empirical distribution of the statistics to approximate the theoretical distribution of the statistics [30], whereas PRODCLIN method is based on the distribution of a product for testing unstandardized indirect effect [31]. An alternative approach is to use maximum likelihood estimate [32]. It is known that likelihood-based confidence interval should capture the asymmetry on the distribution of the indirect effect [29]. Each method has its pros and cons, but we used maximum likelihood method in this study.

The model was modified if necessary. Finally, we identified the best SEM model with the highest goodness-of-fit measure. While there are no golden rules for assessment of model fit, reporting a variety of indices is necessary. Several fit indices including χ^2^, NFI, CFI, GFI and RMSEA can be considered, where NFI is the normed fit index, CFI is the comparative fit index, GFI is the goodness-of-fit index, AGFI is the adjusted goodness-of-fit index, and RMSEA is the root mean square error of approximation [33]. In general, the smaller the χ^2^ value, the better the goodness-of-fit to the data. However, the χ^2^ statistic is sensitive to the samples size and it nearly tends to reject the model when the sample size is large [34]. The other indices are independent of the sample sizes. Cut-offs indicating a good fit for each index are NFI ≥0.95, CFI≥0.95, GFI≥0.95, AGFI≥0.90, and RMSEA≤0.07 [33].

### GWAS data and phenotypic measurements

We analyzed the GWAS data set from the Korea Association Resource (KARE) project. This project was initiated in 2007 in order to undertake a large-scale GWAS. The 10,038 participants were recruited from two community-based cohorts: Ansung, representing a mainly rural community, and Ansan, representing an urban community [35]. After standard quality control procedures for the subjects and SNPs, a total of 8,842 participants and 327,872 SNPs remained. Of them, 4,183 (47.31%) were male and 4,659 (52.69%) were female, with a mean age of 52.22 years (range, 39–70 years). The KARE data include demographic characteristics such as area, sex, and age, as well as multiple phenotypes related to obesity, T2D, and hypertension that were identified based on a review of the participants’ medical records. Our research interests focused on T2D and hypertension as mediated through obesity. Table 1 presents the basic statistics for the data.

**Table 1:** Descriptive statistics of the KARE data.

**Variables**			N (%) or mean±SD
Demographic variables	Sex	Male	4,183 (47.31%)
		Female	4,659 (52.69%)
	Area	Ansung	4,205 (47.56%)
		Ansan	4,637 (52.44%)
	Age		52.22 (8.92)
Intermediate phenotypes	Body mass index, kg/m^2^		24.60 ± 3.12
	Subscapular skin fold thickness, mm		23.69 ± 10.96
	Waist circumference, cm		82.67 ± 8.79
Diseases	Hypertension	Yes	2,393 (27.06%)
		No	6,449 (72.94%)
	T2D	Yes	836 (9.45%)
		No	8,006 (90.55%)
Disease component variables	Systolic blood pressure		117.59 ± 18.28
	Diastolic blood pressure		75.07 ± 11.56
	Fasting blood glucose		87.66 ± 21.88
	Blood glucose after 2 hours		126.76 ± 51.03

KARE, Korea Association Resource; T2D, type 2 diabetes.

We defined T2D using fasting blood glucose (FBG) or blood glucose after 120 minutes (OGTT120), with criteria of FBG ≥ 126 mg/dL, OGTT120 ≥ 200 mg/dL, or the use of antidiabetic medication. Hypertension was defined as systolic blood pressure (SBP) ≥ 140 mm Hg, diastolic blood pressure (DBP) ≥ 90 mm Hg, or the use of antihypertensive medication. As intermediate phenotypes, we considered three traits related to obesity: BMI, WC, and SUB. More specifically, BMI reflects overall body adiposity [36], whereas WC reflects abdominal adiposity (for which visceral adipose tissue is largely responsible), and SUB reflects subcutaneous adiposity [36]. Height (cm), body weight (kg), and waist circumference (cm) were measured using standard methods in light clothes. BMI was calculated as the weight divided by the square of height (kg/m^2^). SUB was measured using a caliper at a vertical fold taken 1 inch below the lowest point of the shoulder blade (mm).

Hypertension was present in 2393 (27.06%) participants, and 836 (9.45%) of participants had T2DM. The average (±SD) values of the obesity-related variables were 24.60 kg/m^2^ (±3.12 kg/m^2^) for BMI, 23.69 mm (± 10.96 mm) for SUB, and 82.7 cm (± 8.79 cm) for WC (Table 1). We analyzed these data using the proposed procedure. From step 1 to step 3, statistical analyses were done using SAS version 9.4 (SAS Corp., Cary, NC, USA). For the SEM analysis in step 4, Lisrel version 9.1 (Scientific software international, Skokie, IL, USA) was used. The threshold for statistical significance was set at α = 0.05, unless otherwise noted.

## Results

### Step 1: Associations between single SNPs and phenotypes

Among the 327,872 SNPs in the KARE dataset, we selected informative SNPs affecting intermediate phenotypes and diseases. We conducted single-SNP analyses using simple linear regression models and logistic regression models for intermediate phenotypes and diseases, respectively. Sex, age, and area were used as covariates. The SNPs were regarded as statistically significant when they showed a p-value less than 1.0E-05 in the single-SNP analysis. The obesity-related phenotypes (BMI, SUB, and WC) were associated with 19 SNPs: 7 SNPs for BMI, 5 SNPs for SUB, and 7 SNPs for WC. For the dichotomous definition of T2D, a single SNP was determined to be significant, with a p-value less than 1.0E-05. For the dichotomous definition of hypertension, 4 SNPs were identified as significant. Finally, we selected 24 SNPs included in one of the 5 SNP blocks. The five SNP blocks consisted of significant SNPs for Sub, BMI, WC, T2D and Hypertension, respectively. Table 2 presents a list of the selected SNPs for each SNP block.

**Table 2:** Significant SNPs for each phenotype through single-SNP analyses.

SNP Block	Traits	SNP	p-value	SNP Block	Traits	SNP	p-value
1	Subscapular skin fold (SUB)	rs17248901	1.92E-07	3	Waist Circumference	rs4667458	4.72E-06
		rs6561930	2.85E-07			rs11933222	9.15E-06
		rs16951883	4.66E-07			rs17178527	2.71E-06
		rs7193144	4.65E-06			rs17092358	6.21E-06
		rs8050136	3.78E-06			rs2074356	7.95E-06
2	BMI	rs527248	2.98E-06			rs17089409	7.07E-06
		rs17178527	2.02E-08			rs17089410	5.67E-06
		rs11000212	1.45E-06	4	T2D	rs11131794	5.09E-06
		rs7193144	3.30E-06	5	Hypertension	rs17249754	3.15E-07
		rs8050136	2.68E-06			rs7136259	4.03E-07
		rs9926289	2.45E-06			rs2254613	6.75E-06
		rs9939609	1.43E-06			rs1378942	8.02E-06

SNP, single-nucleotide polymorphism; T2D, type 2 diabetes.

### Step 2: Construction of latent variables for SNPs

During factor analysis, we investigated the communality between SNPs constructing latent variables. Excluded were three SNPs with very low communality (less than 0.3): rs17178527, which was related to BMI; rs16951883, which was related to SUB; and rs17092358, which was related to WC.

A separate factor analysis was performed for each SNP block to obtain latent variables. Table 3 shows the factor loadings obtained by varimax rotation and variance explained for each phenotype. By using factor loadings, the four SUB-related SNPs in the first SNP block were constructed as two latent variables (hereafter, FACTOR11 and FACTOR12). The six BMI-related SNPs were also constructed as two latent variables (hereafter, FACTOR21 and FACTOR22), and the seven WC-related SNPs were composed of three latent variables, (hereafter, FACTOR31, FACTOR32, and FACTOR33). These latent variables may reflect the common joint action of SNPs on their respective phenotype. The latent variables for the SNPs related to diabetes (hereafter, FACTOR41) and hypertension (hereafter, FACTOR51 and FACTOR52) were also generated in a similar manner. The SNPs classified as corresponding to the same latent variable are shown in square brackets. The two SNPs comprising FACTOR52 have different signs, meaning that they were found to be related in different directions. Both FACTOR11 and FACTOR21 consisted of SNPs from the *FTO* gene, which is known to be associated with fat mass and obesity. The SNPs comprising FACTOR51 were from *ATP2B1*, which has been reported to be a hypertension-related gene [37].

**Table 3:** Latent variable construction of multiple SNPs for each SNP block.

SNP Block	Related phenotype	SNP label	Gene	Factor i1*	Factor i2*	Factor i3*	Variance Explained
1	Subscapular skin fold (SUB)	rs8050136	*FTO*	0.999	-0.001		86.5%
		rs7193144	*FTO*	0.999	-0.003		
		rs17248901	*NBPF21P*	0.004	0.855		
		rs6561930	*LOC100131027*	-0.008	0.855		
2	BMI	rs9926289	*FTO*	0.9982	-0.0057		83.3%
		rs9939609	*FTO*	0.9976	-0.0061		
		rs7193144	*FTO*	0.9973	-0.0072		
		rs8050136	*FTO*	0.9972	-0.0062		
		rs11000212	*DDIT4*	-0.0032	0.7127		
		rs527248	*SEC16B*	-0.0058	0.7115		
3	Waist circumference (WC)	rs17089409	*-*	0.9993	-0.0034	0.0014	67.2%
		rs17089410	*-*	0.9993	-0.0026	0.0010	
		rs17178527	*AK097143*	-0.0174	0.7114	0.1418	
		rs11933222	*-*	0.0128	0.7038	-0.1477	
		rs2074356	*FLJ30092*	0.0046	0.1069	0.7118	
		rs4667458	*-*	-0.0025	-0.1083	0.6794	
4	T2D	rs11131794	*Intergenic*	1.0000			100%
5	Hypertension	rs7136259	*ATP2B1*	0.9849	0.0150		73.7%
		rs17249754	*ATP2B1*	0.9848	0.0141		
		rs1378942	*CSK*	-0.0172	0.7589		
		rs2254613	*MBIP*	-0.0344	-0.6558		

*Factor ij represents the j^th^ latent variable for i^th^ SNP block. SNPs with high factor loading for each latent variable are shown in the same square brackets.

SNP, single-nucleotide polymorphism; BMI, body mass index; T2D, type 2 diabetes.

### Step 3. Investigation of the relationships among variables

We investigated the association among phenotypes by adjusting for the effect of covariates including area, sex, and age. Partial correlation coefficients of the considered phenotypes are presented in Table 4. All three intermediate phenotypes were directly correlated with each other (r>0.547). For hypertension, BMI showed the largest correlation coefficient of the obesity measures (r = 0.194), and WC showed the next largest correlation coefficient (r = 0.180). For T2D, WC showed the largest correlation coefficient (r = 0.122), implying that central body fat may be more closely associated with components of metabolic syndrome, such as T2D and hypertension.

**Table 4:** Partial correlation coefficients among obesity-related phenotypes for T2D and hypertension (n = 8792).

	Subscapular skin fold	BMI	WC	T2D
BMI	0.634***			
WC	0.547***	0.824***		
T2D	0.121***	0.107***	0.122***	
Hypertension	0.135***	0.194***	0.180***	0.102***

*** p<0.0001

T2D, type 2 diabetes; BMI, body mass index; WC, waist circumference.

Before the main SEM analysis, we conducted a path analysis between the intermediate phenotypes and diseases to determine the most proper model. First, we set T2D as a risk factor for hypertension according to recent studies [5, 6]. Second, we established the paths regarding obesity indicators.

As mentioned earlier, we considered three obesity-related phenotypes to capture the various dimensions of obesity including genetic influences on each obesity phenotype: WC reflects abdominal adiposity, BMI reflects overall body adiposity; SUB reflects subcutaneous adiposity [36]. WC is generally known to have a greater association with diabetes or hypertension than the other two obesity indicators. Clinical evidences consistently suggested that the association of insulin resistance (which is thought to be the key common pathway of T2D and hypertension) with WC was stronger than the corresponding associations of insulin resistance with BMI or SUB [36, 38]. In our analysis, WC also showed the strongest correlation with T2D, and BMI or SUB showed considerable correlation with WC. Therefore, we have established the ordered paths to maximize the clinical relevance, where WC directly affects the diseases, and BMI or SUB affect diseases through WC. We considered the paths in which BMI or SUB directly affect T2D or hypertension independently of WC.

**Fig 3:**
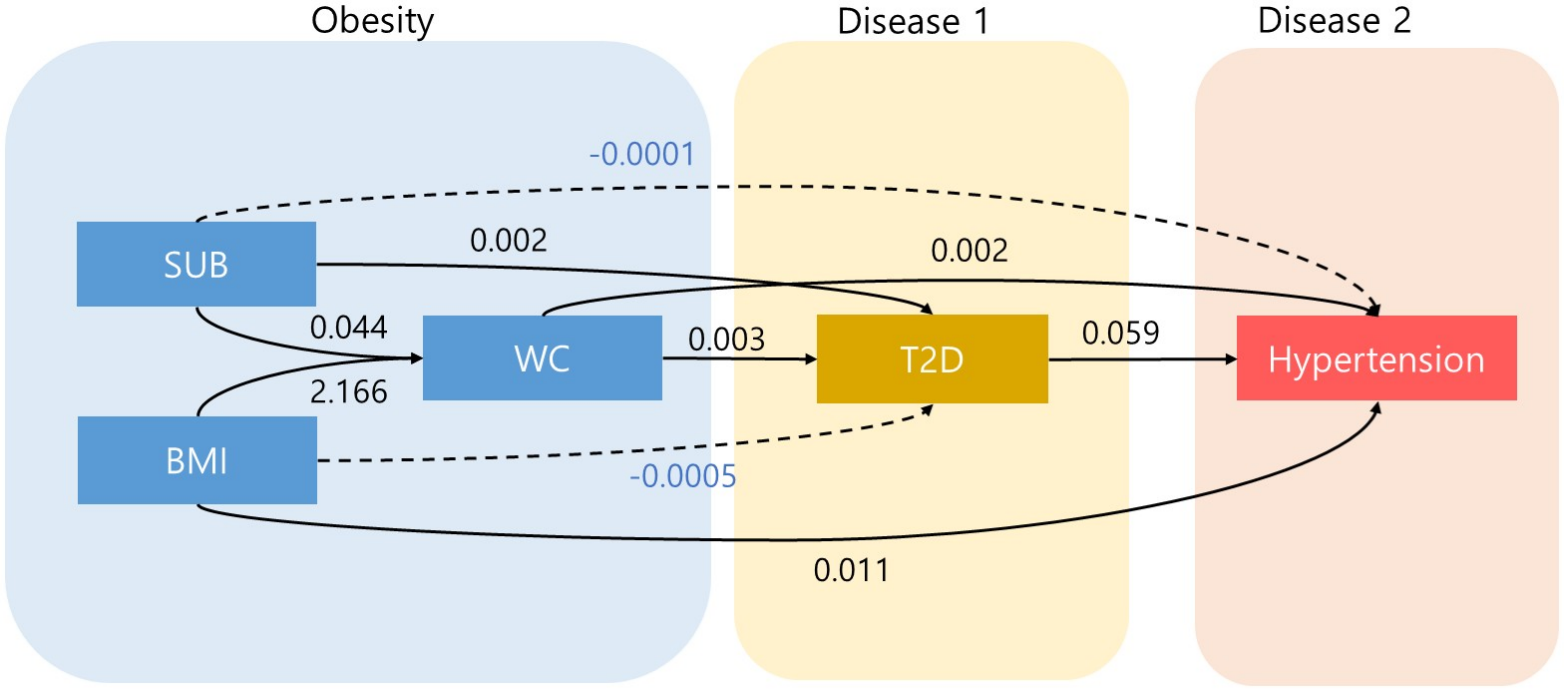
Path diagram and direct effect estimates from the path analysis of phenotypes. Solid lines indicate that effects were statistically significant (p<0.05), whereas dotted lines present statistically insignificant effects.

As a result, we established the path from obesity to T2D and hypertension that is shown in Fig 3. It displays the coefficient estimates of the path analysis, which indicate the strength of the effect of the independent variable on the dependent variable. Paths with solid lines indicate that effects were statistically significant (p<0.05), whereas two paths with dotted lines showed statistically insignificant effects.

### Step 4: Structural equation model of multiple SNPs and multiple phenotypes

The SEM was constructed based on the joint action of the multiple SNPs and multiple intermediate phenotypes identified in the previous steps. By developing an SEM, we reflected the relationships between all SNPs, their joint action or latent variables, obesity-related phenotypes, and their possible morbidity (i.e., their effect on diseases). In hypothesized model construction, we assumed that the error terms were uncorrelated, which was an important assumption for causal inference in performing mediation analysis. Model coefficients of SEM were estimated by maximum likelihood method using the sample covariance matrix of KARE data, with sex, age, and area as covariates, yielding consistent and efficient estimates. The fit of the hypothesized SEM was improved by allowing measurement errors correlated for rs9926289 and rs9939609, rs7193144 and rs8050136, rs7193144 and rs7193144, rs8050136 and rs8050136. Model diagnosis confirmed that the maximum likelihood estimation method was appropriate. Under the proposed SEM, we considered the causal relationships among endogenous latent variables of WC and BMI. Correlations between latent variables for each phenotype were not large enough to cause multicollinearity between latent variables.

Through the modification process, we identified the best SEM model based on various goodness-of-fit measures (χ^2^ = 536.52, NFI = 0.997, CFI = 0.998, GFI = 0.995, AGFI = 0.993, RMSEA = 0.012). These indices indicated that the resulting SEM should have high goodness-of-fit.

**Fig 4:**
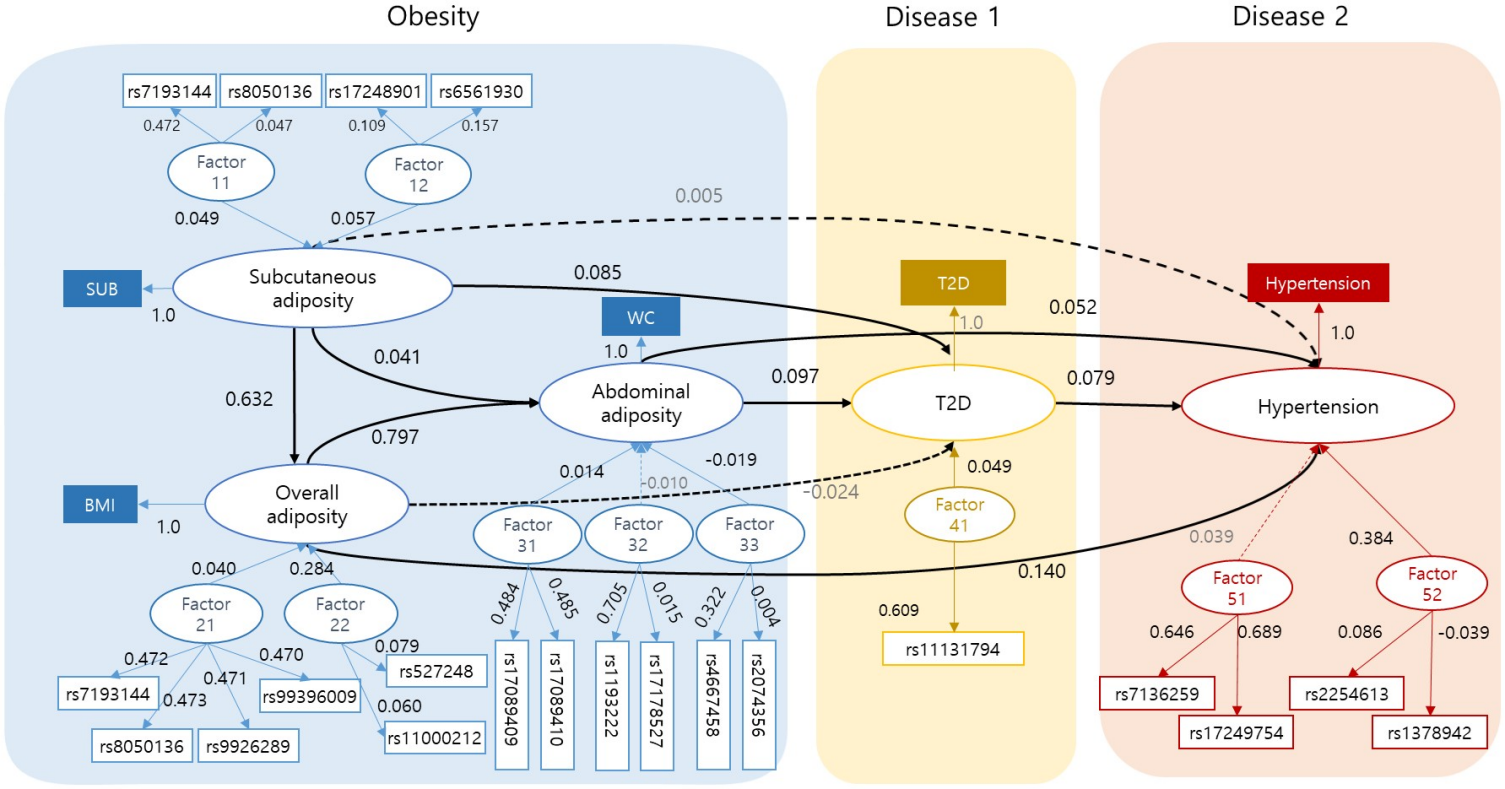
Path diagram and direct effect estimates of the final SEM. Factor ij represents the j^th^ latent variable for i^th^ SNP block. Solid lines indicate that effects were statistically significant (p<0.05), whereas dotted lines present statistically insignificant effects.

Fig 4 and Table 5 show the standardized effects of the final SEM. In the analysis of causal relationships among intermediate phenotypes and diseases, subcutaneous adiposity showed a direct, statistically significant relationship for overall adiposity (b = 0.632, t = 76.25), abdominal adiposity (b = 0.041, t = 5.23) and T2D (b = 0.085, t = 6.25), but statistical significance was not reached for hypertension (b = 0.01, t = 0.285). Overall adiposity directly affected abdominal adiposity (b = 0.797, t = 101.79), T2D (b = -0.024, t = -2.10), and hypertension (b = 0.140, t = 4.59). Abdominal adiposity directly affected T2D (b = 0.097, t = 4.61) and hypertension (b = 0.052, t = 7.44). Solid lines in Fig 4 indicate that effects were statistically significant (p<0.05), whereas dotted lines present statistically insignificant effects.

**Table 5:** Direct, indirect, and total effects of the final SEM.

Endogenous variables	Exogenous variables	Direct effect		Indirect effect		Total effect	
		Estimate	SE	Estimate	SE	Estimate	SE
Subcutaneous adiposity	FACTOR11	0.049***	0.011			0.049***	0.011
	FACTOR12	0.057***	0.011			0.057***	0.011
Overall adiposity	FACTOR11			0.031***	0.007	0.031***	0.007
	FACTOR12			0.036***	0.007	0.036***	0.007
	FACTOR21	0.041**	0.021			0.040**	0.021
	FACTOR22	0.284***	0.055			0.284***	0.055
	Subcutaneous adiposity	0.632***	0.008			0.632***	0.008
Abdominal adiposity	FACTOR11			0.027***	0.006	0.027***	0.006
	FACTOR12			0.031***	0.006	0.031***	0.006
	FACTOR21			0.032**	0.016	0.032**	0.016
	FACTOR22			0.226***	0.044	0.226***	0.044
	FACTOR31	0.014***	0.006			0.014***	0.006
	FACTOR32	-0.010	0.006			-0.010	0.006
	FACTOR33	-0.019***	0.006			-0.019***	0.006
	Subcutaneous adiposity	0.041***	0.008	0.503***	0.008	0.544***	0.009
	Overall adiposity	0.797***	0.008			0.797***	0.008
T2D	FACTOR11			0.006***	0.001	0.006***	0.001
	FACTOR12			0.007***	0.001	0.007***	0.001
	FACTOR21			0.002*	0.001	0.002*	0.001
	FACTOR22			0.015***	0.005	0.015***	0.005
	FACTOR31			0.001**	0.001	0.001**	0.001
	FACTOR32			-0.001	0.001	-0.001	0.001
	FACTOR33			-0.002***	0.001	-0.002***	0.001
	FACTOR41	0.049***	0.011			0.049***	0.011
	Subcutaneous adiposity	0.085***	0.014	0.038***	0.009	0.123***	0.011
	Overall adiposity	-0.024*	0.020	0.077***	0.015	0.053***	0.014
	Abdominal adiposity	0.097***	0.019			0.097***	0.019
Hypertension	FACTOR11			0.007***	0.002	0.007***	0.002
	FACTOR12			0.008***	0.002	0.008***	0.002
	FACTOR21			0.008*	0.004	0.008*	0.004
	FACTOR22			0.053***	0.013	0.053***	0.013
	FACTOR31			0.001*	0.000	0.001*	0.000
	FACTOR32			-0.001	0.000	-0.001	0.000
	FACTOR33			-0.001***	0.001	-0.001***	0.001
	FACTOR41			0.004***	0.001	0.004***	0.001
	FACTOR51	0.039	0.068			0.039	0.068
	FACTOR52	0.384***	0.162			0.384***	0.162
	Subcutaneous adiposity	0.005	0.020	0.127***	0.017	0.132***	0.011
	Overall adiposity	0.140***	0.031	0.046***	0.015	0.186***	0.027
	Abdominal adiposity	0.052***	0.011	0.008***	0.002	0.060***	0.018
	T2D	0.079***	0.011			0.079***	0.011

* p< 0.10

** p< 0.05

*** p<0.01

SEM, structural equation model; SE, standard error; T2D, type 2 diabetes.

In contrast, in the analysis of genetic factors, information from 17 SNPs was used to predict T2D and information from 21 SNPs was used to predict hypertension. FACTOR11 and FACTOR12, which were latent variables consisting of SUB-related SNPs, showed significant direct effects on subcutaneous adiposity. They also had significant indirect effects on overall adiposity, abdominal adiposity, T2D, and hypertension. Similarly, the latent variables from the SNPs related to BMI (FACTOR21 and FACTOR22) affected overall adiposity directly and affected abdominal adiposity indirectly. FACTOR21 also significantly affected T2D and hypertension indirectly. Of the latent variables from the SNPs related to WC, FACTOR31 and FACTOR33 showed significant relationships with abdominal adiposity and T2D, but FACTOR32 did not. Of the WC-related latent variables, only FACTOR33 showed significance for hypertension. One of the latent variables from the hypertension-related SNPs (FACTOR52) was significantly associated with hypertension, but the other (FACTOR51) was not. However, although FACTOR51 consisted of SNPs that had significant individual effects on hypertension in the single-SNP analysis, the latent variables were no longer significant when the various factors were considered together.

Integrating these observations, T2D was found to be significantly affected by subcutaneous adiposity, overall adiposity, and abdominal adiposity, and all the latent variables from the SNPs except FACTOR32. The largest direct effect on T2D came from abdominal adiposity (b = 0.097, t = 5.21). However, including indirect effects, subcutaneous adiposity had the largest total effect (b = 0.123, t = 11.60). Meanwhile, hypertension was directly and indirectly associated with overall adiposity, abdominal adiposity, and T2D. Subcutaneous adiposity significantly affected hypertension indirectly, but not directly. The largest effect on hypertension was obtained for FACTOR52 (b = 0.384, t = 2.37), and overall adiposity showed the second largest direct effect (b = 0.186, t = 6.88). The results were similar when only direct effects were considered.

## Discussion

Hypertension and T2D are important public health concerns, as the prevalence of each is increasing worldwide [39, 40]. The coexistence of hypertension and T2D dramatically increases the risk (2- to 4-fold) of cardiovascular disease and all-cause death [4]. Although studies have investigated the effects of obesity-related factors on T2D and hypertension separately, few studies have investigated the pathways underlying hypertension through obesity-related traits.

In this study, we aimed to improve our understanding of the pathways underlying hypertension and T2D driven by genetic variants and obesity-related traits by conducting a multivariate analysis. In order to achieve these goals, we developed an analytical process consisting of four steps that yielded successful results. In step 1, we investigated GWAS variants that affected intermediate phenotypes and disease, the first research goal. In step 2, we found communalities or similarities among SNPs for each phenotype, the second research goal. Step 3, the path analysis of multiple intermediate phenotypes and diseases, suggested plausible associations among traits, the third research goal. Step 4 enabled us to achieve our final research goal through an SEM analysis of the associations among multiple SNPs, multiple phenotypes, and multiple diseases. Conclusively we developed a quantitative map simultaneously showing the relationships among GWAS variants, intermediate phenotypes, T2D, and hypertension.

This analysis provides insights into the mechanisms underlying T2D and hypertension. Our findings highlight the importance of subcutaneous adiposity and abdominal adiposity, as well as latent variables from SNPs, as driving elements of T2D in the Korean population. The impacts of latent variables of the SNPs, overall adiposity, abdominal adiposity, and T2D on hypertension were also confirmed. The resulting model had high goodness-of-fit measures.

## References

[1] Organization, W.H., *Global health risks*: *mortality and burden of disease attributable to selected major risks*. 2009: Geneva: World Health Organization.

[2] Lozano R., et al, Global and regional mortality from 235 causes of death for 20 age groups in 1990 and 2010: a systematic analysis for the Global Burden of Disease Study 2010. The lancet, 2012 380(9859): p. 2095–2128. 10.1016/S0140-6736(12)61728-0 PMC10790329 23245604

[3] De Boer I.H., et al, Diabetes and hypertension: a position statement by the American Diabetes Association. Diabetes Care, 2017 40(9): p. 1273–1284. 10.2337/dci17-0026 28830958

[4] Ferrannini E. and Cushman W.C., Diabetes and hypertension: the bad companions. The Lancet, 2012 380(9841): p. 601–610. 10.1016/S0140-6736(12)60987-8 22883509

[5] Tsimihodimos V., et al, Hypertension and diabetes mellitus: coprediction and time trajectories. Hypertension, 2018 71(3): p. 422–428. 10.1161/HYPERTENSIONAHA.117.10546 29335249 PMC5877818

[6] Sun D., et al, Type 2 Diabetes and Hypertension: A Study on Bidirectional Causality. Circulation research, 2019.

[7] Cheung B.M. and Li C., Diabetes and hypertension: is there a common metabolic pathway? Current atherosclerosis reports, 2012 14(2): p. 160–166. 10.1007/s11883-012-0227-2 22281657 PMC3314178

[8] Karns R., et al, Modeling metabolic syndrome through structural equations of metabolic traits, comorbid diseases, and GWAS variants. Obesity (Silver Spring), 2013 21(12): p. E745–54. 23512735 10.1002/oby.20445

[9] Visscher P.M., et al, Five years of GWAS discovery. Am J Hum Genet, 2012 90(1): p. 7–24. 10.1016/j.ajhg.2011.11.029 22243964 PMC3257326

[10] Ng F.L., Warren H.R., and Caulfield M.J., Hypertension genomics and cardiovascular prevention. Annals of translational medicine, 2018 6(15): p. 291–291. 10.21037/atm.2018.06.34 30211179 PMC6123211

[11] Mohlke K.L. and Boehnke M., Recent advances in understanding the genetic architecture of type 2 diabetes. Human Molecular Genetics, 2015 24(R1): p. R85–R92. 10.1093/hmg/ddv264 26160912 PMC4572004

[12] Taylor J.Y., et al, An overview of the genomics of metabolic syndrome. J Nurs Scholarsh, 2013 45(1): p. 52–9. 10.1111/j.1547-5069.2012.01484.x 23368731 PMC3594572

[13] VanderWeele T.J., Mediation Analysis: A Practitioner’s Guide. Annual Review of Public Health, 2016 37(1): p. 17–32. 10.1146/annurev-publhealth-032315-021402 26653405

[14] Agler R. and De Boeck P., On the Interpretation and Use of Mediation: Multiple Perspectives on Mediation Analysis. Front Psychol, 2017 8: p. 1984 10.3389/fpsyg.2017.01984 29187828 PMC5694788

[15] Gunzler D., et al, Introduction to mediation analysis with structural equation modeling. Shanghai archives of psychiatry, 2013 25(6): p. 390–394. 24991183 10.3969/j.issn.1002-0829.2013.06.009 PMC4054581

[16] Stein C., Morris N., and Nock N., *Structural Equation Modeling*. Vol. 850 2012 495–512. 10.1007/978-1-61779-555-8_27 22307716

[17] Hox J. and Bechger T., *An Introduction to Structural Equation Modeling*. Vol. 11 1999.

[18] MacCallum R.C. and Austin J.T., Applications of Structural Equation Modeling in Psychological Research. Annual Review of Psychology, 2000 51(1): p. 201–226. 10.1146/annurev.psych.51.1.201 10751970

[19] Stegmann G., *Review of A Beginner’s Guide to Structural Equation Modeling (4th ed*.*)*, *by Randall E*. *Schumacker & Richard G*. *Lomax*: *New York*, *NY*: *Routledge*, *2016*. *351 pp*. *$65*.*91 (paperback)*. Vol. 24 2017 475–477.

[20] Li R., et al, Structural model analysis of multiple quantitative traits. PLoS Genet, 2006 2(7): p. e114 10.1371/journal.pgen.0020114 16848643 PMC1513264

[21] Rosa Guilherme JM, V. B.D., Gustavo de los Campos, Wu Xiao-Lin, Gianola Daniel, Silva Martinho A Inferring causal phenotype networks using structural equation models. Genetics Selection Evolution, 2011 43(6): p. 13. 10.1186/1297-9686-43-6 PMC3056759 21310061

[22] Kim J., et al, Application of Structural Equation Models to Genome-wide Association Analysis. Genomics Inform, 2010 8(3): p. 150–158.

[23] Nuzhdin S.V., Friesen M.L., and McIntyre L.M., Genotype-phenotype mapping in a post-GWAS world. Trends Genet, 2012 28(9): p. 421–6. 10.1016/j.tig.2012.06.003 22818580 PMC3476940

[24] Verhulst B., Maes H.H., and Neale M.C., GW-SEM: A Statistical Package to Conduct Genome-Wide Structural Equation Modeling. Behavior genetics, 2017 47(3): p. 345–359. 10.1007/s10519-017-9842-6 28299468 PMC5423544

[25] Stein C.M., et al, Structural equation model-based genome scan for the metabolic syndrome. BMC Genet, 2003 4 Suppl 1: p. S99. 14975167 10.1186/1471-2156-4-S1-S99 PMC1866540

[26] Song Y.E., Morris N.J., and Stein C.M., Structural equation modeling with latent variables for longitudinal blood pressure traits using general pedigrees. BMC Proc, 2016 10(Suppl 7): p. 303–307. 10.1186/s12919-016-0047-4 27980653 PMC5133482

[27] Jesús Rosel I.P., Longitudinal Data Analysis with Structural Equations. European Journal of Research Methods for the Behavioral and Social Sciences, 2008 4: p. 37–50.

[28] Osborne J., *What is Rotating in Exploratory Factor Analysis*? Vol. 20 2015 1–8.

[29] Cheung M.W.L., Comparison of methods for constructing confidence intervals of standardized indirect effects. Behavior Research Methods, 2009 41(2): p. 425–438. 10.3758/BRM.41.2.425 19363183

[30] Shrout P.E. and Bolger N., Mediation in experimental and nonexperimental studies: New procedures and recommendations. Psychological Methods, 2002 7(4): p. 422–445. 12530702

[31] MacKinnon D.P., et al, Distribution of the product confidence limits for the indirect effect: program PRODCLIN. Behavior research methods, 2007 39(3): p. 384–389. 17958149 10.3758/bf03193007 PMC2819369

[32] Leth-Steensen C. and Gallitto E., Testing Mediation in Structural Equation Modeling: The Effectiveness of the Test of Joint Significance. Educational and psychological measurement, 2016 76(2): p. 339–351. 10.1177/0013164415593777 29795869 PMC5965588

[33] Hooper D., Coughlan J., Mullen M., Structural Equation Modelling: Guidelines for Determining Model Fit. Electronic Journal of Business Research Methods, 2008 6(1): p. 8.

[34] Barrett P., Structural equation modelling: Adjudging model fit. Personality and Individual Differences, 2007 42(5): p. 815–824.

[35] Cho Y.S., et al, A large-scale genome-wide association study of Asian populations uncovers genetic factors influencing eight quantitative traits. Nat Genet, 2009 41(5): p. 527–34. 10.1038/ng.357 19396169

[36] Snijder M., et al, What aspects of body fat are particularly hazardous and how do we measure them? International Journal of Epidemiology, 2005 35(1): p. 83–92. 10.1093/ije/dyi253 16339600

[37] Levy D., et al, Genome-wide association study of blood pressure and hypertension. Nat Genet, 2009 41(6): p. 677–87. 10.1038/ng.384 19430479 PMC2998712

[38] Vazquez G., et al, Comparison of Body Mass Index, Waist Circumference, and Waist/Hip Ratio in Predicting Incident Diabetes: A Meta-Analysis. Epidemiologic Reviews, 2007 29(1): p. 115–128. 17494056 10.1093/epirev/mxm008

[39] Cho N., et al, IDF Diabetes Atlas: Global estimates of diabetes prevalence for 2017 and projections for 2045. Diabetes research and clinical practice, 2018 138: p. 271–281. 10.1016/j.diabres.2018.02.023 29496507

[40] Forouzanfar M.H., et al, Global burden of hypertension and systolic blood pressure of at least 110 to 115 mm Hg, 1990–2015. Jama, 2017 317(2): p. 165–182. 10.1001/jama.2016.19043 28097354

